# Supplementary material for: Smooth Interpolating Curves with Local Control and Monotone Alternating Curvature
Source: Comput Graph Forum. 2022 Oct 6;41(5):25–38. doi: 10.1111/cgf.14600 (PMC9827861; doi:10.1111/cgf.14600)
Supplement: Supplementary file 1 — Supplement Material [file CGF-41-25-s001.zip › Local-Smooth-Interpolating-MonoCurvature/extern/clothoids/submodules/Utils/ThirdParties/Eigen/eigen-3.4.0.zip!/eigen-3.4.0/bench/perf_monitoring/resources/chart_footer.html]

 /\* setup the chart and its options \*/
var chart = nv.models.lineChart()
.color(d3.scale.category10().range())
.margin({left: 75, bottom: 100})
.forceX([0]).forceY([0]);
chart.x(function(datum){ return datum.r; })
.xAxis.options({
axisLabel: customSettings.XLABEL || 'Changeset',
tickFormat: d3.format('.0f')
});
chart.xAxis
.tickValues(changesets\_count)
.tickFormat(function(d){return changesets[d]})
.rotateLabels(-90);
chart.y(function(datum){ return datum.v; })
.yAxis.options({
axisLabel: customSettings.YLABEL || 'GFlops'/\*,
tickFormat: function(val){ return d3.format('.0f')(val) + ' GFlops'; }\*/
});
chart.tooltip.headerFormatter(function(d) { return changesets[d]
+ '

'
+ changesets\_details[d] + "

"; });
//chart.useInteractiveGuideline(true);
d3.select('#chart').datum(data).call(chart);
var plot = d3.select('#chart > g');
/\* setup the title \*/
plot.append('text')
.style('font-size', '24px')
.attr('text-anchor', 'middle').attr('x', '50%').attr('y', '20px')
.text(customSettings.TITLE || '');
/\* ensure the chart is responsive \*/
nv.utils.windowResize(chart.update);
